# Primary resistance to afatinib in a patient with NSCLC harboring *EGFR* p.E709_T710delinsD and brain metastases: a case report and literature review

**DOI:** 10.3389/fonc.2026.1981577

**Published:** 2026-10-26

**Authors:** Su-Su Dong, Xuan Zhang, Wen Dong

**Affiliations:** 1 Department of Respiratory Medicine, Changde Hospital, Xiangya School of Medicine, Central South University (The First People’s Hospital of Changde City), Changde, Hunan, China; 2 Department of Oncology, Changde Hospital, Xiangya School of Medicine, Central South University (The First People’s Hospital of Changde City), Changde, Hunan, China

**Keywords:** brain metastases, *EGFR* p.E709_T710delinsD, non-small cell lung cancer, PACC mutation, primary afatinib resistance

## Abstract

*EGFR* p.E709_T710delinsD is a rare in-frame deletion-insertion P-loop and alpha-C-helix compressing (PACC) mutation of *EGFR* exon 18. Current evidence suggests that patients with non-small cell lung cancer (NSCLC) harboring this variant generally respond to afatinib. However, primary resistance to afatinib in this molecular subset is rarely reported. We report a 68-year-old man diagnosed with stage IV lung adenocarcinoma harboring an *EGFR* p.E709_T710delinsD mutation (variant allele frequency, 45.9%) with co-occurring EGFR amplification (fold change 1.5). Brain metastases were present at baseline. The patient was treated with oral afatinib at an initial dose of 30 mg once daily but the disease progressed rapidly, suggesting possible primary targeted resistance. The patient died 5 weeks later. This case represents a rare example of primary resistance to afatinib in non-small cell lung cancer (NSCLC) harboring the EGFR p.E709_T710delinsD mutation. Given the lack of tissue and molecular evidence to confirm the resistance mechanism in this case, this report combines previous literature to explore the potential factors associated with treatment failure and their clinical implications. This case does not refute the overall sensitivity of this variant to afatinib, but suggests that in patients with high baseline disease burden and aggressive tumor biology, this variant may present as a phenotype with no initial benefit and rapid progression. For such rapidly progressive cases, early radiographic reassessment should be performed, and repeat biopsy or circulating tumor DNA (ctDNA) testing should be pursued as soon as safely feasible to clarify the resistance mechanism and guide subsequent treatment.

## 1 Introduction

Epidermal growth factor receptor (*EGFR*) mutations influence treatment decisions in patients with non-small cell lung cancer (NSCLC). Targeted therapy with EGFR tyrosine kinase inhibitors (TKIs) is a standard modality of the first-line treatments for stage III or stage IV NSCLC patients with *EGFR* mutations and greatly improves their prognosis (1). *EGFR* mutations include common and rare mutations. Exon 19 deletion (19del) and the exon 21 point mutation L858R account for 80–90% of EGFR mutations observed in NSCLC and are generally sensitive to EGFR TKIs (2). The remaining 10–20% of *EGFR* mutations in NSCLC comprise rare mutations, including point mutations, deletions, and insertions within exons 18–25 of the *EGFR* gene (3). *EGFR* exon 18 mutations are an uncommon driver gene variant subtype, among which the *EGFR* p.E709_T710delinsD mutation is a rare in-frame deletion-insertion P-loop and alpha-C-helix compressing (PACC) mutation (4). Existing clinical evidence is primarily derived from case reports, small-sample cohorts, and *in vitro* studies; therefore, its therapeutic sensitivity may remain uncertain. Most available data suggest that this variant is more sensitive to second-generation EGFR-TKIs (particularly afatinib) than to first-generation EGFR-TKIs. However, treatment responses in reported cases have been variable. A few case reports have shown that NSCLC with the *EGFR* p.E709_T710delinsD mutation was sensitive to afatinib (5). However, Zeng (6)described *EGFR* p.E709_T710delinsD as a potential acquired resistance mechanism to afatinib in patients with E709H lung adenocarcinoma. A tertiary-center audit of primary and secondary resistance also listed *EGFR* p.E709_T710delinsD among cases with primary resistance to first- and/or second-generation EGFR-TKIs, with a table comment noting no response to afatinib (7). These data suggest that responses to afatinib in patients with *EGFR* p.E709_T710delinsD may not be uniform: available reports are dominated by clinical benefit, although isolated clues suggestive of possible primary insensitivity have also been reported.

Herein, we report a patient with advanced lung adenocarcinoma and brain metastases harboring *EGFR* p.E709_T710delinsD. Despite first-line afatinib at an initial dose of 30 mg, the patient failed to achieve effective disease control, and experienced rapid disease progression followed by death within a short period. This case does not negate the evidence that *EGFR* p.E709_T710delinsD is generally sensitive to afatinib. rather, it suggests that, in high-risk settings such as brain metastases, this variant may manifest as a phenotype characterized by lack of initial benefit, rapid disease progression, and poor outcome. Given the absence of tissue and molecular evidence to confirm a resistance mechanism in this case, we discuss potential contributing factors to treatment failure and the clinical implications of this case in the context of previous reports.

## 2 Case presentation

A 68-year-old male smoker was admitted to the hospital because of a cough, expectoration, blood in sputum for 1 year, and headache and an unsteady gait for 1 month. The patient had developed a paroxysmal cough with production of bloodstained sputum 1 year previously but had not experienced chest pain or shortness of breath and did not seek medical treatment at the time. One month previously, he had developed headache, fatigue, blurred vision, an unsteady gait, and pain in the right shoulder, which led him to visit the hospital. His other medical history was unremarkable.

On examination, he had no palpable superficial lymph nodes, and his muscle strength and tone were normal. Pulmonary auscultation revealed decreased breath sounds in the left lower lung, without dry or wet rales. Laboratory tests revealed elevated levels of the tumor markers, carcinoembryonic antigen (CEA) (995.15 ng/mL; normal <5 ng/mL), cytokeratin 19 fragments (CYFRA21-1) (21.99 ng/mL; normal <3.3 ng/mL) and neuron-specific enolase (NSE) (45.02 ng/mL; normal <17 ng/mL), but the squamous cell carcinoma antigen (SCCA) level was within the normal range (1.10 ng/mL; normal <2.7 ng/mL). The patient’s complete blood count, electrolytes, fasting blood glucose, lipid profile, coagulation function, myocardial enzymes, and liver and kidney function were normal. Chest computed tomography confirmed a lung mass and pleural effusion in the left lung (**Figure 1A**). Histopathological analysis of tissue biopsy samples obtained from the lower lobe of the left lung via bronchoscopy revealed adenocarcinoma. The immunohistochemistry results revealed CK7(+), TTF-1(+), napsin A(+), CK5/6(−), P40(−), CD56(−), Syn(−), ALK(D5F3)(−), and Ki67(+20%). Brain magnetic resonance imaging showed brain metastases (**Figure 1B**). Next-generation sequencing (NGS) was performed on the lung biopsy tissue (GenePlus Medical Laboratory). *EGFR* p.E709_T710delinsD was detected at a variant allele frequency (VAF) of 45.9%, accompanied by *EGFR* amplification with a fold change (fc) of 1.5. Based on the pathology-assessed tumor content of the specimen (10%), purity correction was applied to estimate the tumor-cell gene copy number (GCN): GCN = (fc × 2 − 2)/tumor fraction + 2, yielding a GCN of 12. T790M, *ALK*, *ROS1*, *MET*, *BRAF*, *HER2*, *RET*, *NTRK1*, and *KRAS* were all negative. In addition, the expression of programmed death-ligand 1(PD-L1) was relatively low (combined positive score [CPS] <1, tumor proportion score [TPS] <1%).

**Figure 1:**
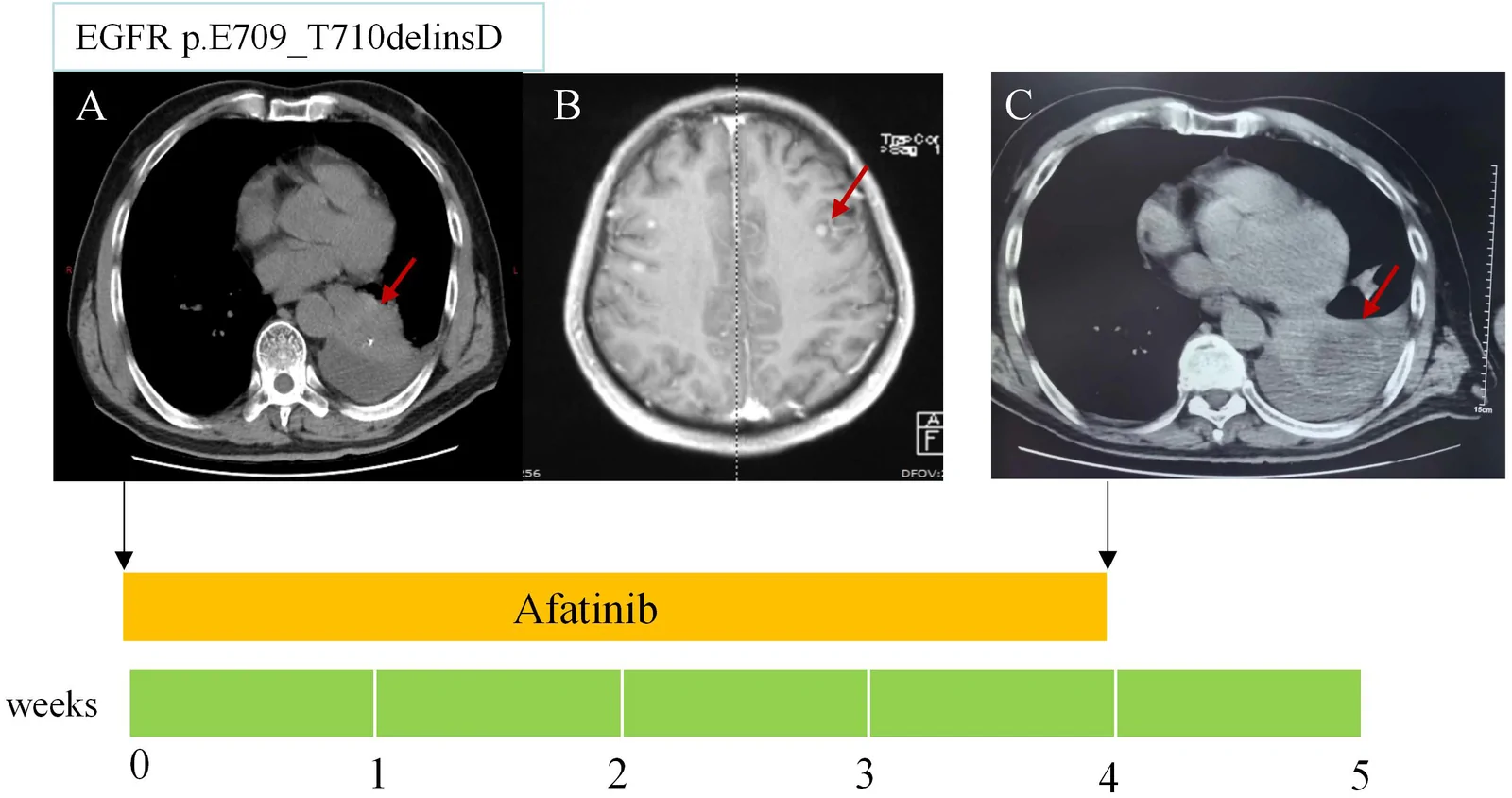
The patient’s clinical course illustrated using lung computed tomography (CT) and brain magnetic resonance imaging (MRI). **(A)** Baseline chest CT showing a mass in the left lower lobe of the lung and a left pleural effusion. **(B)** Brain MRI showing brain metastases. **(C)** Chest CT 4 weeks after starting afatinib treatment showing disease progression.

Based on these findings, the patient was diagnosed with cT2N2M1c (stage IV) lung adenocarcinoma according to the International Association for the Study of Lung Cancer (IASLC) and the American Joint Committee on Cancer (AJCC) 8th TNM classification. The patient had an Eastern Cooperative Oncology Group (ECOG) Performance Status (PS) score of 2 and declined chemotherapy. Several case reports have shown that the patients with adenocarcinoma harboring the *EGFR* p.E709_T710delinsD mutation can respond to afatinib (5, 8–15). The patient received afatinib at a starting dose of 30 mg/day without experiencing adverse reactions such as diarrhea or rash. However, his clinical condition did not improve. Follow-up chest CT 4 weeks after starting afatinib revealed disease progression (**Figure 1C**), and his general condition continued to deteriorate, with his ECOG PS score increasing to 4. He was unable to cooperate with contrast-enhanced brain MRI due to his poor clinical condition. Furthermore, the patient declined all subsequent treatments and further diagnostic evaluations. He died approximately 1 week after treatment discontinuation. Family consent for autopsy was not obtained; therefore, the exact cause of death remains unknown.

## 3 Discussion

In recent years, at least 23 cases of NSCLC with the *EGFR* p.E709_T710delinsD have been reported with documentation of their response to EGFR TKIs (including gefitinib, erlotinib, osimertinib, dacomitinib, and afatinib) (5, 8–23) (**Table 1**). First-generation EGFR TKIs are relatively ineffective for treating NSCLC with this mutation. Six patients treated with gefitinib had a median progression-free survival (PFS) of 2.7 months (16, 17). Seven patients treated with erlotinib had a median PFS of 3.5 months (5, 17–21). The sensitivity of second-generation TKIs may be higher than that of first-generation TKIs. One patient achieved a partial response (PR) to treatment with dacomitinib, with a PFS of 7 months (22). The effectiveness of afatinib against this mutation has been reported in 9 cases, with a median PFS of 11 months (5, 8–15). The effectiveness of osimertinib against this mutation has been reported in only three cases. One patient received osimertinib as first-line therapy, but showed suboptimal results, with a PFS of 9 weeks (23). Two patients achieved partial remission with afatinib treatment, but as the disease progressed were treated with second-line osimertinib, which failed to control the disease (12, 15). Further clinical data are needed to define the activity of osimertinib in patients harboring EGFR p.E709_T710delinsD.

**Table 1:** Summary of case reports of cases of patients with non-small cell lung cancer harboring *EGFR* p.E709_T710delinsD.

Patient no.	Reference	Age (years)	Sex	Smoking status	Histology	Stage	BM	TKI(s) used (line)	PFS (months)	OS (months)
1	(5)	63	M	NA	ADC	NA	No	Erlotinib (3^rd^), afatinib (4^th^)	NA	NA
2	(8)	52	F	Never	ADC	IV	Yes	Afatinib (1^st^)	NA	NA
3	(9)	56	F	Never	ADC	IV	No	Afatinib (6^th^)	7	NA
4	(10)	71	M	NA	ADC	III	Yes	Afatinib (3^rd^)	11	>21
5	(11)	57	F	Never	ADC	III	No	Afatinib (2^nd^)	12	36
6	(12)	78	F	Never	ADC	IV	No	Afatinib (1^st^), osimertinib (2^nd^)	11	12
7	(13)	57	F	Never	ADC	IV	No	Afatinib (1^st^)	>17	>17
8	(14)	70	F	Never	ADC	II	No	Afatinib (1^st^), almonertinib (2^nd^)	22	>27
9	(15)	43	M	Yes	ADC	IV	Yes	Afatinib (1^st^), osimertinib (2^nd^)	5	NA
10	(16)	61	F	Never	ADC	IV	NA	Gefitinib (NA)	5.1	79
11	(16)	65	M	Yes	ADC	IV	NA	Gefitinib (NA)	0.9	11.1
12	(16)	57	F	Never	ADC	IV	NA	Gefitinib (NA)	0.6	24.1
13	(16)	79	M	Yes	ADC	IV	NA	Gefitinib (NA)	6.2	6.2
14	(16)	68	M	Yes	ADC	IV	NA	Gefitinib (NA)	2.3	29.5
15	(17)	66	F	Yes	ADC	IV	NA	Gefitinib (1^st^)	3	24
16	(17)	46	F	Yes	ADC	II	NA	Erlotinib (2^nd^)	4	26
17	(17)	57	F	Never	ADC	IV	NA	Erlotinib (2^nd^)	3	18
18	(18)	NA	NA	NA	NA	IV	NA	Erlotinib (1^st^)	8	NA
19	(19)	60	M	Never	ADC	IV	NA	Erlotinib (NA)	1	3
20	(20)	50	F	Never	NSCLC	III/IV	NA	Erlotinib (2^nd^)	1.3	1.7
21	(21)	88	F	Never	ADC	IV	No	Erlotinib (1^st^)	6	NA
22	(22)	56	F	Never	ADC	IV	No	Dacomitinib (2^nd^)	7	NA
23	(23)	70	F	Yes	ADC	IV	No	Osimertinib (1^st^)	2.1	NA
24	This case	68	M	Yes	ADC	IV	Yes	Afatinib (1^st^)	1	1.1

ADC, adenocarcinoma; BM, brain metastasis; NA, not available; no., number; NSCLC, non-small cell lung cancer; OS, overall survival; PFS, progression-free survival; Ref, reference; TKI: tyrosine kinase inhibitor.

In the cases summarized in this article, nine patients with *EGFR* p.E709_T710delinsD who received 30 mg or 40 mg afatinib achieved partial response. Additionally, an audit of primary and secondary resistance by Suryavanshi listed *EGFR* p.E709_T710delinsD among cases associated with primary resistance to first- and/or second-generation EGFR-TKIs and noted in a table comment that this patient had no response to afatinib (7). This case was excluded from the summarized cases in this article due to the absence of data on staging, metastatic profile, presence of brain metastases, afatinib dose and line of therapy, whether afatinib was used as monotherapy, sequence of afatinib and chemotherapy administration. In contrast to the favorable outcomes reported in the aforementioned cases, this patient exhibited early disease progression following first-line afatinib therapy. It is important to acknowledge that this patient presented with multiple adverse prognostic factors at baseline, including ECOG PS 2, brain metastases, and markedly elevated CEA (995.15 ng/mL), which likely contributed substantially to the rapid disease progression and early mortality within five weeks.

Considering adverse-event risks including severe diarrhea and rash, as well as the patient’s general clinical condition (ECOG PS 2) at that time, the patient was initiated on afatinib at a reduced dose of 30 mg daily. Currently, evidence on the prognostic impact of afatinib dose in patients with advanced *EGFR*-mutant NSCLC and brain metastases is limited to a few retrospective studies, and the findings are conflicting. Tan (24) reported that, patients with baseline brain metastases who started on 40 mg had superior median PFS compared to those who started on 30 mg (13.3 vs. 5.3 months; HR 0.39). However, Wei et al. found that in patients with baseline brain metastases receiving afatinib monotherapy, there was no statistically significant difference in the intracranial objective response rate between the initial daily dose of 30 mg and 40 mg groups (86.7% vs. 80.0%; P = 0.581) (25). Hung et al. reported that, among patients with compound or dual rare *EGFR* mutations, afatinib at 30 mg provided efficacy comparable to the 40 mg dose, with better tolerability (26). However, to date, there have been no comparative studies of afatinib dosing specifically in patients with *EGFR* p.E709_T710delinsD, particularly in those with brain metastases. Therefore, treatment failure with 30 mg in this case cannot address whether increasing the dose to 40 mg might improve outcomes; likewise, previously reported successful cases treated with 30 mg are insufficient to establish that drug exposure was adequate in this patient. In the absence of data on plasma drug concentrations, fasting/postprandial status, and drug interactions, insufficient exposure cannot be completely excluded. Because post-treatment brain MRI was not completed, intracranial response in this case should be considered not evaluable; therefore, concurrent intracranial progression cannot be confirmed, CNS progression cannot be established as a possible cause of treatment failure, and its contribution to clinical deterioration cannot be fully excluded.

NGS revealed an *EGFR* fold change of 1.5. Based on the pathology-assessed tumor content (10%), the testing laboratory estimated a tumor-cell gene copy number (GCN) of approximately 12 after purity correction and interpreted the finding as *EGFR* amplification; FISH validation was not performed. Of note, clinical benefit from afatinib has been reported in the patient harboring E709_T710delinsD with concomitant *EGFR* amplification (14). Therefore, *EGFR* amplification may not account for the rapid disease progression observed in this case.

The treatment failure in this case cannot exclude the possibility of intrinsic insensitivity of the variant itself. nine reported patients with *EGFR* p.E709_T710delinsD responded to afatinib. however, due to the limited sample size, the true response rate remains unclear. Even for *EGFR*-sensitizing mutations with the strongest evidence, the objective response rate to first-line TKIs is approximately 70% (27). In an analysis of 197 patients with TKI-sensitive *EGFR* mutations, Lee et al. reported that 11 patients (5.6%) experienced primary resistance (defined as disease progression within 3 months of TKI initiation); only three of them harbored coexisting genetic alterations that might explain the resistance (T790M, *MET* amplification, and *ALK* fusion), while some patients had no detectable molecular explanation (28). Therefore, the treatment failure in this case is not contradictory to the reported sensitivity, and our patient may represent a poorly responding subgroup of this variant.

Small cell histological transformation can render EGFR-TKIs completely ineffective, and its rapid progression resembles the clinical course of the present case. However, transformation usually occurs after prolonged TKI exposure; the shortest reported interval is 3 months after TKI initiation (29). In our patient, primary progression occurred only 4 weeks after treatment initiation, making this mechanism unlikely. Nevertheless, given the absence of a second biopsy, transformation cannot be completely excluded.

This case does not imply that *EGFR* p.E709_T710delinsD is generally insensitive to afatinib; rather, it suggests that patients harboring this rare variant may present with a phenotype of no initial benefit. For patients with a high baseline disease burden and strongly aggressive tumor biology, earlier clinical and radiological re-evaluation should be considered if symptoms deteriorate rapidly and imaging assessment at conventional time points cannot be completed; re-biopsy of the progressing lesion or plasma ctDNA testing should be pursued as soon as safely feasible to clarify the resistance mechanism and guide subsequent treatment.

This case study has several limitations. This report is based on a single-case observation without re-biopsy or autopsy. Treatment adherence and actual drug exposure could not be verified retrospectively. The EGFR copy number was a purity-corrected estimate based on a tumor content of 10% (which was at the lower limit of the assay’s quality control threshold), and FISH validation was not performed.

## Data Availability

The raw data supporting the conclusions of this article will be made available by the authors, without undue reservation.
